# ATP-binding cassette family C member 1 constrains metabolic responses to high-fat diet in male mice

**DOI:** 10.1530/JOE-24-0024

**Published:** 2024-07-03

**Authors:** Elisa Villalobos, Allende Miguelez-Crespo, Ruth A Morgan, Lisa Ivatt, Mhairi Paul, Joanna P Simpson, Natalie Z M Homer, Dominic Kurian, Judit Aguilar, Rachel A Kline, Thomas M Wishart, Nicholas M Morton, Roland H Stimson, Ruth Andrew, Brian R Walker, Mark Nixon

**Affiliations:** 1 University/British Heart Foundation Centre for Cardiovascular Science, The Queen’s Medical Research Institute, University of Edinburgh, Edinburgh, United Kingdom; 2 Translational and Clinical Research Institute, Newcastle University, Newcastle upon Tyne, United Kingdom; 3 Scotland’s Rural College, The Roslin Institute, Easter Bush Campus, United Kingdom; 4 The Roslin Institute, Royal (Dick) School of Veterinary Studies, College of Medicine and Veterinary Medicine, University of Edinburgh, Easter Bush Campus, Edinburgh, United Kingdom; 5 Centre for Systems Health and Integrated Metabolic Research, Nottingham Trent University, Nottingham, United Kingdom

**Keywords:** glucocorticoids, steroids, transport, metabolism, obesity, homeostasis

## Abstract

Glucocorticoids modulate glucose homeostasis, acting on metabolically active tissues such as liver, skeletal muscle, and adipose tissue. Intracellular regulation of glucocorticoid action in adipose tissue impacts metabolic responses to obesity. ATP-binding cassette family C member 1 (ABCC1) is a transmembrane glucocorticoid transporter known to limit the accumulation of exogenously administered corticosterone in adipose tissue. However, the role of ABCC1 in the regulation of endogenous glucocorticoid action and its impact on fuel metabolism has not been studied. Here, we investigate the impact of *Abcc1* deficiency on glucocorticoid action and high-fat-diet (HFD)-induced obesity. In lean male mice, deficiency of *Abcc1* increased endogenous corticosterone levels in skeletal muscle and adipose tissue but did not impact insulin sensitivity. In contrast, *Abcc1*-deficient male mice on HFD displayed impaired glucose and insulin tolerance, and fasting hyperinsulinaemia, without alterations in tissue corticosterone levels. Proteomics and bulk RNA sequencing revealed that *Abcc1* deficiency amplified the transcriptional response to an obesogenic diet in adipose tissue but not in skeletal muscle. Moreover, *Abcc1* deficiency impairs key signalling pathways related to glucose metabolism in both skeletal muscle and adipose tissue, in particular those related to OXPHOS machinery and Glut4. Together, our results highlight a role for ABCC1 in regulating glucose homeostasis, demonstrating diet-dependent effects that are not associated with altered tissue glucocorticoid concentrations.

## Introduction

Glucocorticoids are required to maintain glucose and lipid homeostasis in times of physiological stress, ensuring an adequate fuel supply for the body (Kuo *et al.* 2015). In key metabolic tissues such as adipose tissue, skeletal muscle and liver, glucocorticoids act to prevent glycolysis and instead promote gluconeogenesis (Kuo *et al.* 2013). However, chronic glucocorticoid excess, e.g. in Cushing’s syndrome, causes metabolic dysfunction including hyperglycaemia and obesity (Pivonello *et al.* 2016, Kaikaew *et al.* 2019). Moreover, increased tissue levels of glucocorticoids have been described in humans with obesity and in animal models of obesity (Anderson *et al.* 2016, Pivonello *et al.* 2016, Luijten *et al.* 2019). In particular, increased adipose (Rask *et al.* 2001, Kershaw *et al.* 2005, Gathercole *et al.* 2007) or skeletal muscle (Trost *et al.* 2002, Morgan *et al.* 2009, Loerz & Maser 2017) exposure to glucocorticoids is accompanied by insulin resistance, consistent with the effects of glucocorticoids on glucose and lipid utilisation.

Controlling intracellular levels of glucocorticoids within target tissues can influence glucocorticoid levels over and above their regulation by the hypothalamic–pituitary–adrenal (HPA) axis. Studies using strategies to reduce glucocorticoid levels in tissues, e.g. by inhibition of 11-β-hydroxysteroid dehydrogenase type 1 (11β-HSD1), showed prevention of weight gain and metabolic dysfunction in animal models of obesity and in humans (Bray *et al.* 1992, Anderson *et al.* 2016). However, 11β-HSD1 inhibitors have not progressed beyond phase II trials due to their insufficient efficacy on metabolic outcomes (Rosenstock *et al.* 2010, Scott *et al.* 2014). We have also recently shown that mice with deficiency of carbonyl reductase 1 (*Cbr1*), a further glucocorticoid regulator in adipose tissue, display lower levels of fasting glucose and improved glucose tolerance (Bell *et al.* 2021). A key question is whether there are other mechanisms that confer tissue-specific control of glucocorticoid levels that might be important in obesity and tractable to therapy.

ATP-binding cassette subfamily C member 1 (ABCC1) is a multidrug efflux transporter present in the plasma membrane of many cell types, including adipocytes and myotubes, but not hepatocytes (Ling *et al.* 2014, Qu *et al.* 2014, Sajja & Cucullo 2015, Shi *et al.* 2017). *ABCC1* is highly expressed in adipose tissue and skeletal muscle, but poorly expressed in the liver (Devine *et al.* 2023). While primarily studied due to its capacity to efflux drugs and its role in determining resistance to chemotherapeutic agents in cancer (Ling *et al.* 2014, Shi *et al.* 2017, Bernal-Sore *et al.* 2018, Mohankumar *et al.* 2018), we recently identified ABCC1 as a regulator of HPA axis negative feedback in humans (Kyle *et al.* 2022) and as a glucocorticoid transporter in white adipose tissue (Nixon *et al.* 2016). Mice with a global deletion of *Abcc1* or mice administered the ABCC1 inhibitor, probenecid, accumulate exogenously administered corticosterone within adipose tissue, associated with exaggerated subcutaneous adipose glucocorticoid-responsive gene transcription. Moreover, analysis of *ABCC1* expression revealed an increase in mRNA levels in subcutaneous and visceral adipose tissue from obese patients compared with lean controls (Nixon *et al.* 2016), suggesting a compensatory mechanism to ‘protect’ adipose from glucocorticoid excess. Additionally, recent evidence demonstrates relatively high levels of *ABCC1* in skeletal muscle in humans and mice (Nixon *et al.* 2016, Devine *et al.* 2023), indicating a potential role for this efflux transporter in regulating glucocorticoid action in key non-adipose metabolic tissues. Importantly, the role of *Abcc1* in murine models of obesity has not yet been described.

We tested the hypothesis that mice lacking *Abcc1* exhibit an adverse metabolic profile due to increased intra-tissue endogenous glucocorticoid action in white adipose tissue and skeletal muscle. Using a global knockout of *Abcc1* in adult male mice, we aimed to evaluate the influence of *Abcc1* on the metabolic profile in both lean and obese conditions, using control chow and high-fat diet (HFD), respectively.

## Materials and methods

### Animals

Male *Abcc1* knockout (*Abcc1*-KO*)* mice were purchased from The Jackson Laboratory (B6.129S1-Abcc1tm1Acs/VoreJ, Stock no.: 028129) and bred in-house with female C57BL/6J (Stock no.: 000664). *Abcc1^−/−^* (KO) and *Abcc1^+/+^* (WT) mice were generated from heterozygous crosses. Mice were born at expected Mendelian ratios and were genotyped by PCR analysis of genomic ear clip DNA using specific primers flanking exon 3 and part of exon 2 (P1: GTTTGAGCCACTCTCTCTGG, P2: GTGTTAAGCCGATGAGCAATC, and P3: CCTTCTATCGCCTTCTTGACG) as described previously (Lorico *et al.* 1997, Nixon *et al.* 2016).

All experiments were performed in adult (>8-week-old) male mice. Mice were maintained in individually ventilated cages in groups (3−5) at 21˚C with a 12 h light:12 h darkness cycle (lights on from 07:00 h to 19:00 h). Food and water were available *ad libitum.* Mice were administered either control (chow) diet (2.71% kcal from fat, RM1(E) 801002, Special Diet Services) or HFD (58% kcal from fat plus sucrose, D12331, Research Diets) for up to 9 weeks. Body weight was measured weekly at the same time of day in each group (AM). Mice were culled between 09:00 h and 11:30 h by decapitation to minimise the stress response (<1 min between handling and decapitation). Trunk blood was collected in EDTA-coated microcentrifuge tubes and subjected to centrifugation (10,000 ***g***, 5 min) to obtain plasma. All the procedures were performed under a UK Home Office licence and approved by the University of Edinburgh, Bioresearch & Veterinary Services.

### Physiological measurements

Insulin and glucose tolerance tests were performed in animals following a 6-h fast (09:00–15:00 h) during week 7 and 8 of the diet, respectively. For the insulin tolerance test (ITT), insulin (0.75 U/kg; cat. no. I9278, Sigma-Aldrich) was administered by i.p. injection, and blood glucose was measured by glucometer (Accu-Chek) in samples from tail venesection at 15, 30, 60, 90 and 120 min post injection. For the glucose tolerance test (GTT), glucose (1 g/kg; cat. no. 50-99-7, Sigma-Aldrich) was administered by i.p. injection, and blood glucose was measured by glucometer as above at 15, 30, 60, 90 and 120 min post injection. Fasting insulin levels were assessed on plasma samples obtained at the ‘0-min’ time point of ITT and quantified by ELISA (Merck, EZRMI-13K). A diurnal sampling of blood for glucocorticoid profiling was performed (08:00 h and 20:00 h) on conscious, unstressed mice (<1 min between handling and collection) by tail venesection and collected in EDTA-coated capillary blood tubes (Microvette), prior to analysis of steroids in plasma by liquid chromatography–tandem mass spectrometry (LC-MS/MS). ACTH levels were evaluated on plasma samples (trunk blood) by ELISA assay (MD Bioproducts, M046006). Homeostatic model assessment of insulin resistance (HOMA-IR) was calculated using the formula (fasting insulin (mg/dL) × fasting glucose (mmol/L))/22.5.

### Western blotting

Tissue protein lysates were prepared in RIPA lysis extraction buffer, supplemented with Halt, phosphatase and protease inhibitors (Thermo Scientific). Samples were disrupted using a TissueLyser II (Qiagen) and 5 mm stainless-steel beads (30 Hz, three cycles of 15 s). Protein concentration was quantified using a bicinchoninic acid (BCA) assay (Thermo Scientific). Extracted proteins (25 µg) were resolved by SDS-PAGE, using Criterion TGX Precast Protein Gels 4–20% (Bio-Rad) under reducing and denaturing conditions. Proteins were transferred to nitrocellulose membranes using the Trans-Blot Turbo Blotting System (Bio-Rad). Membranes were blocked with 5% skimmed milk (Scientific Laboratory Supplies) in Tris-buffered saline and then subjected to Western blotting using antibodies against OXPHOS proteins (ab110413, dilution 1:1000) and GLUT4 (MA1-83191, dilution 1:1000). Primary antibodies were used at the described dilutions in 3% BSA (Sigma-Aldrich) in Tris-buffered saline with Tween 20 and incubated overnight (4°C). Secondary antibodies (IRDye 800CW or IRDye 680CW (LI-COR) (anti-mouse, rat and rabbit IgGs) were used at 1:10,000 dilutions in 3% BSA solution in Tris-buffered saline and incubated for 1 h at room temperature. Total protein staining was performed using Revert(LI-COR, 926-11011). Detection of protein was performed using an Odyssey CLx Imaging system (LI-COR). Densitometric analyses were performed using Image StudioSoftware (LI-COR).

### RNA isolation and quantitative RT-PCR

Tissue disruption was carried out using a TissueLyser II (Qiagen) and 5 mm stainless-steel beads (30 Hz, three cycles of 15 s). Total RNA was extracted from tissues using Aurum Total RNA Fatty and Fibrous Tissue Kit (Bio-Rad). Contaminating DNA was removed by treating the samples with DNase I (Bio-Rad). About 500 ng RNA was used for reverse transcription using iScript reagent (Bio-Rad), and the products were analysed by qRT-PCR (LightCycler 480, Roche). The qRT-PCR was performed using Sybr Green (iTaq, Bio-Rad). Primer sequences are provided in Table 1. All primers were previously calibrated and used at efficiencies between 90% and 110%. Data analysis was performed using the Pfaffl method (Pfaffl 2001).

**Table 1:** Forward and reverse primer sequences for qRT-PCR.

Target mRNA	Accession	Forward primer (5′–3′)	Reverse primer (5′–3′)
*Per1*	NM_001159367.2	AACGGGATGTGTTTCGGGGTGC	AGGACCTCCTCTGATTCGGCAG
*Abcc1*	NM_001425178.1	GGAATTTTCGGCTGAGTGTC	AGCCAAATATTGCTGCACCT
*Fkbp5*	NM_010220.4	GAGCTTATGTACGAGGTCACCC	GCGTGTACTTGCCTCCCTTG
*Redd1*	NM_029083.2	GGTCTGCAGCCAGAGAAGAG	TCCAGGTATGAGGAGTCTTCC
*Actb*^a^	NM_007393.5	CACTGTCGAGTCGCGTCC	TCATCCATGGCGAACTGGTG
*Hprt*^a^	NM_013556.2	AAGCCTAAGATGAGCGCAAG	TTACTAGGCAGATGGCCACA

^a^Housekeeping genes.

### Steroid profiling by liquid chromatography–tandem mass spectrometry

Glucocorticoids (corticosterone and 11-dehydrocorticosterone) were quantified in plasma derived from trunk blood and tail venesection, and in tissues using an adapted protocol previously described (Bell *et al.* 2021). Briefly, steroid extraction was performed in samples of plasma (10 µL diurnal sampling and 100 µL trunk blood samples), liver (80–100 mg), gastrocnemius (40–50 mg) and sWAT (50–80 mg). Tissues were homogenised (Bead Ruptor Elite Bead Mill Homogenizer, Omni International) in acetonitrile with formic acid 0.1%. A calibration standard curve (0.0025–500 ng/mL) was prepared and run alongside the samples. Homogenates were centrifugated and filtered through a Biotage Filter+ 96-well plate (0.22 µm), after which all samples and standards were enriched with d8-corticosterone as the internal standard. The homogenate was then extracted using an ISOLUTE PLD+ 96-well plate, for tissue samples (Biotage, Uppsala, Sweden) and a Microsolute SLE 200 plate, for plasma (Biotage), and eluted under positive pressure. Extracts were dried down under nitrogen at 40°C and re-suspended for analysis by LC-MS/MS using a Waters I-Class UPLC connected to a QTrap 6500+ Mass Spectrometer (AB Sciex). Standards and samples were injected (20 μL) onto a Kinetex C18 column (150 × 2.1 mm and 2.6 μm; Phenomenex, #TN-1063) fitted with a 0.5 μm Ultra KrudKatcher (Phenomenex, #00F-4783-AN) at a flow rate of 0.3 mL/min. The mobile phase system comprised water with 0.05 mM ammonium fluoride and methanol with 0.05 mM ammonium fluoride. The mass spectrometer was operated in positive ion electrospray ionisation mode using multiple reaction monitoring of steroids and internal standards. The instrumentation was operated using Analyst 1.6.3 (AB Sciex) and quantitative analysis of the data was carried out by least squares regression of the peak area ratio of the steroid to the corresponding internal standard with equal or 1/× weighting using MultiQuant software v3.0.3 (AB Sciex).

### Bulk RNA sequencing analysis

Samples (*n* = 4 per experimental group) of gastrocnemius muscle and adipose tissue (sWAT) were processed for RNA isolation (as above). Total RNA was quantified using a Nanodrop spectrophotometer (Thermo Scientific), and integrity was assessed using an Agilent 2100 Bioanalyzer (Agilent Technologies Inc.) and the Agilent RNA 6000 Nano kit. Library preparation and transcriptome sequencing were conducted by Novogene Co. Ltd. cDNA libraries were sequenced using the Illumina NovaSeq platform (Illumina Inc.). Bioinformatic analysis was performed by Fios Genomics (Edinburgh, UK). The quality of the data was assessed by the FastQC control tool. Reads were aligned to a mouse reference genome build GRCm39 using the STAR aligner, followed by calculation of alignment and mapping statistics. At least 87% of read pairs were uniquely mapped to one region of the genome. Analysis was performed using log2 fold of change (FC), calculated individually for each comparison, for example, between WT and *Abcc1*-deficient mice under chow diet, or between chow and HFD in WT mice. Volcano plots for single comparisons are presented as unadjusted *P*-values (Agudelo *et al.* 2018). Further analyses were performed correcting for multiple testing (FDR adjusted) with a significance threshold of *P* < 0.05.

### Proteomics

Samples (*n* = 4 per experimental group) of gastrocnemius muscle (50–120 mg) and subcutaneous white adipose tissue (70–230 mg) were homogenised in an extraction buffer (5% SDS in 50 mM triethylammonium bicarbonate buffer, pH 8.5) at a sample to buffer ratio of 1:10 (w/v) using a Precellys homogeniser (5000 rpm – 2 × 10 sec) with beads in a ceramic vial (Precellys Lysing Kit, Tissue homogenizing CK mix). Following homogenisation, samples were centrifuged for 10 min at 16,000 ***g***, and the supernatant was transferred into a clean low protein-binding vial. Subsequently, the supernatant was sonicated for ten cycles with 30 s on and 30 s off per cycle (Pico Sonicator Diagenode bioruptor). After sonication, samples were centrifuged (16,000 ***g*** for 10 min), and the supernatant was collected. A BCA assay was performed.

The proteins were reduced with dithiothreitol and alkylated with iodoacetamide prior to tryptic digestion on S-TRAP (Protifi) cartridges following the manufacturer’s protocol. The resulting peptides were cleaned up using C18 stagetips. Purified peptides were separated over a 90-minute gradient on an Aurora-25 cm column (IonOpticks) using an UltiMate RSLCnano LC System (Dionex) coupled to a timsTOF FleX mass spectrometer via a CaptiveSpray ionisation source. The gradient was delivered at a flow rate of 200 nL/min and washout was performed at 500 nL/min. The column temperature was set at 50°C. For DDA-PASEF acquisition, full scans were recorded from 100 to 1700 *m/z* spanning from 1.45 to 0.65 Vs/cm^2^ in the mobility (1/K0) dimension. Up to ten PASEF MS/MS frames were performed on ion-mobility separated precursors, excluding singly charged ions which are fully segregated in the mobility dimension, with a threshold and target intensity of 1750 and 14,500 counts, respectively. Raw mass spectral data was processed using PEAKS Studio X-Pro Software (Bioinformatics Ltd). Searches were performed against the Uniprot mouse sequence database with an MS1 precursor tolerance of 20 ppm and MS2 tolerance of 0.06 Da. Full tryptic digestion allowing one missed cleavage, fixed modification of cysteine [+57.02], and oxidation of methionine and deamination of asparagine and glutamine were also specified for the database search. Label-free quantitative analysis (LFQ) was performed with default parameters and with optional identification. Analysis was performed using log2 fold of change (FC), calculated individually for each comparison, for example, between WT and *Abcc1*-deficient mice with a chow diet (Karagianni *et al.* 2022). Separate comparisons per dietary condition were performed to generate distinct DEP lists for subsequent ingenuity pathway analysis (Qiagen) (Agudelo *et al.* 2018). Volcano plots were generated using VolcaNoseR (Goedhart & Luijsterburg 2020).

### Statistical analysis

Results are expressed as mean ± s.e.m. Sample size (*n* = 12 per group) was calculated based on the variability of GTTs previously performed in C57Bl/6J mice under chow and HFD conditions to detect differences of 20% (*α* = 0.05 and power = 90%) in blood glucose. Downstream analyses (biomolecular) were performed on a representative subset of the samples (*n* = 3–8), as detailed in each legend. Analyses were performed using GraphPad Prism (Version 9.2.0, 2021 GraphPad Software LLC.). Comparisons between WT and KO mice on different diets were by two-way ANOVA, followed by a *post hoc* test (Tukey). Comparisons of measurements over time were performed by two-way ANOVA with repeated measures, followed by a *post hoc* test (Sidak). The normal distribution of the samples was assessed by the Shapiro–Wilk test (*P* > 0.05), and if the dataset did not have a normal distribution, a non-parametric test was used (Mann–Whitney *U* test or Kruskal–Wallis). Differences with a *P*-value < 0.05 were considered statistically significant.

## Results

### In lean mice, *Abcc1* deficiency does not impact weight gain, glucose tolerance or insulin resistance

**Figure 1:**
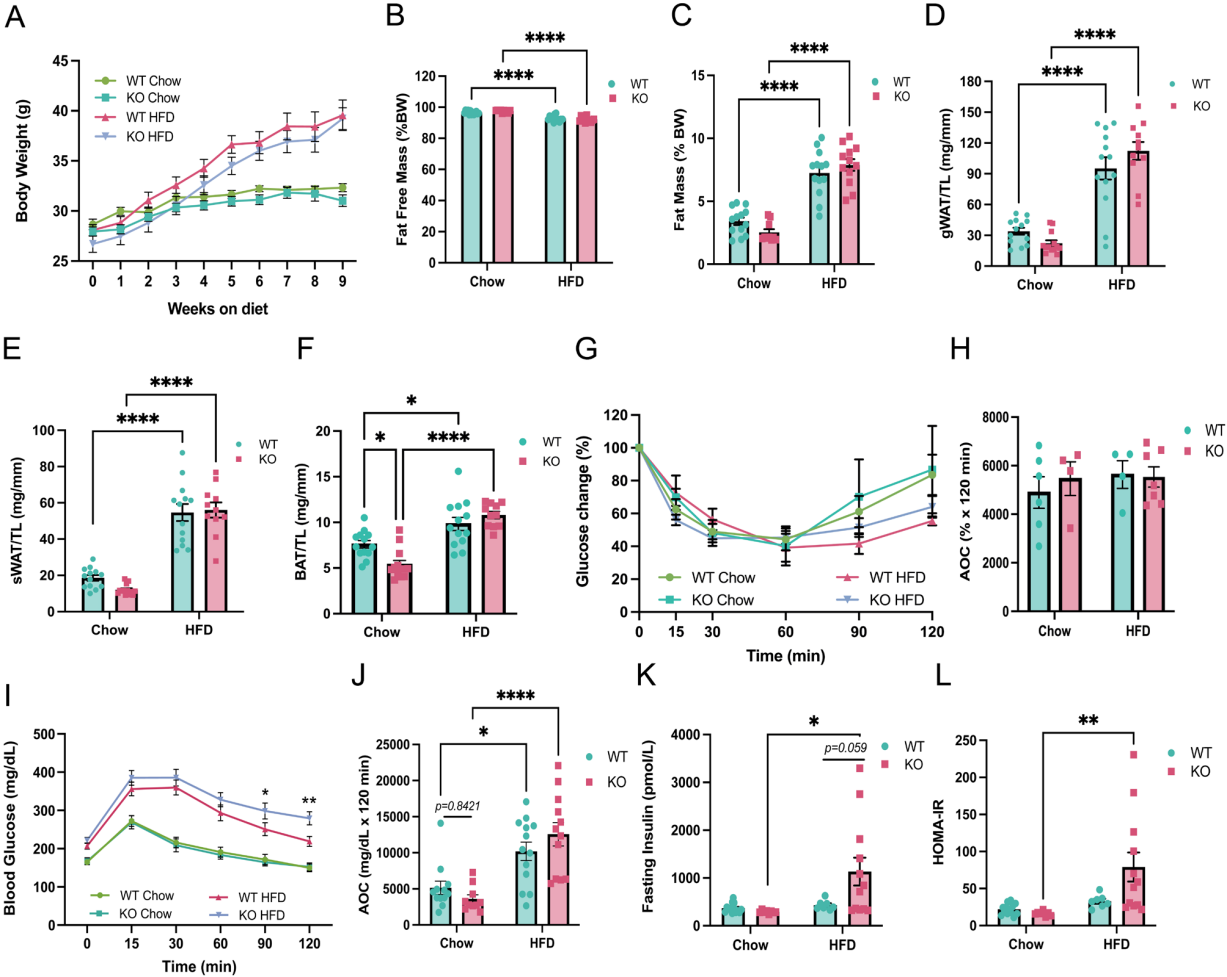
Comparison of metabolic profile between age-matched *Abcc1*-deficient mice (KO) and wild-type littermates (WT) mice shows amplified insulin resistance in Abcc1 KO on high-fat diet (HFD). Animals (12–13 per group) were fed either a chow diet or HFD feeding started at 8 to 10 weeks of age and continued for 9 weeks. (A) Body weight, measured weekly, in WT and KO male mice while receiving chow and HFD. Data were analysed by a mixed-effect model with Holm–Sidak’s multiple comparisons tests (time: *P* < 0.01, experimental group: *P* < 0.01, interaction: *P* < 0.01). (B) Percentage (%) of fat-free mass (diet: *P* < 0.01, genotype: *P* = 0.77, interaction: *P* = 0.06) and fat mass (diet: *P* < 0.01, genotype: *P* = 0.77, interaction: *P* = 0.06) (C), with respect to total body weight, at the end of the study. (D) Weight of gonadal white adipose tissue (gWAT), (E) subcutaneous white adipose tissue (sWAT), and (F) brown adipose tissue (BAT), normalised by the length of the tibia (TL) (diet: *P* < 0.01, genotype: *P* = 0.23, interaction: *P* < 0.01). Metabolic tests were performed after 7–8 weeks of chow or HFD, after 5 h of fast. (G) Intraperitoneal insulin tolerance test (IP-ITT) performed after 7 weeks of dietary intervention in WT and KO mice, results shown as glucose change (percentage) from baseline (time: *P* < 0.01, experimental group: *P* = 0.67, interaction: *P* = 0.10), and (H) quantification of area over the curve (AOC) (diet: *P* = 0.50, genotype: *P* = 0.69, interaction: *P* = 0.57), (*n* = 4–7 animals per group). (I) Intraperitoneal glucose tolerance test (IP-GTT) performed after 8 weeks of dietary intervention in WT and KO mice (time: *P* < 0.01, experimental group: *P* < 0.01, interaction: *P* < 0.01), and (J) quantification of area over the curve (AOC) (diet: *P* < 0.01, genotype: *P* = 0.73, interaction: *P* = 0.11) (*n* = 8–13 animals per group). (K) Fasting insulin levels in WT and KO mice after 7 weeks of dietary intervention (diet: *P* = 0.01, genotype: *P* = 0.09, interaction: *P* = 0.04). (L) Homeostatic model assessment for insulin resistance (HOMA-IR) (diet: *P* < 0.01, genotype: *P* = 0.11, interaction: *P* = 0.04). *P < 0.05, **P < 0.01, ***P < 0.001, ****P < 0.0001 by repeated measures ANOVA (A) and two-way ANOVA with Tukey’s multiple comparisons test. Data are expressed as mean ± s.e.m.

To evaluate the role of *Abcc1* on the metabolic phenotype, *Abcc1* KO male mice (8–12 weeks old) and wild-type (WT) littermates were fed either a chow diet or HFD (58% kcal fat w/ sucrose) for 9 weeks. Mice with *Abcc1* deficiency did not differ in body weight from WT mice when receiving a chow diet (Fig. 1A). Moreover, fat-free mass and fat mass (gonadal and subcutaneous WAT) were not significantly different in KO (Fig. 1B, C, D, and E) compared to WT mice. However, brown adipose tissue (BAT) mass was significantly lower in KO vs WT mice (Fig. 1F). Both insulin tolerance (Fig. 1G, H, Supplementary Fig. 1B and C, see section on supplementary materials given at the end of this article) and glucose tolerance (Fig. 1I and J) were similar between KO and WT mice on the chow diet. Furthermore, fasting insulin (Fig. 1K) and fasting glucose levels (Supplementary Fig. 1A) were not significantly different between genotypes.

### In diet-induced obesity, *Abcc1* deficiency exacerbates glucose intolerance and insulin resistance

To determine the influence of HFD on *Abcc1*, we assessed mRNA levels in WAT and skeletal muscle. Our results showed similar levels of *Abcc1* in WT mice in chow versus HFD conditions in both tissues (Supplementary Fig. 1C and D).

Under HFD-fed conditions, there were no differences between genotypes in measures of fat mass (Fig. 1C) or adipose depot tissue weights, including BAT (Fig. 1D, E and F). Insulin tolerance remained similar in WT and KO mice (Fig. 1G and H), but glucose intolerance with HFD was exacerbated in KO mice (Fig. 1I and J). While fasting glucose was not different between genotypes (Supplementary Fig. 1A), fasting insulin levels (Fig. 1K), and HOMA-IR (Fig. 1L) were significantly elevated in KO vs WT mice under HFD conditions.

### *Abcc1* deficiency increases tissue corticosterone in lean but not obese mice, and not independently of circulating levels

**Figure 2:**
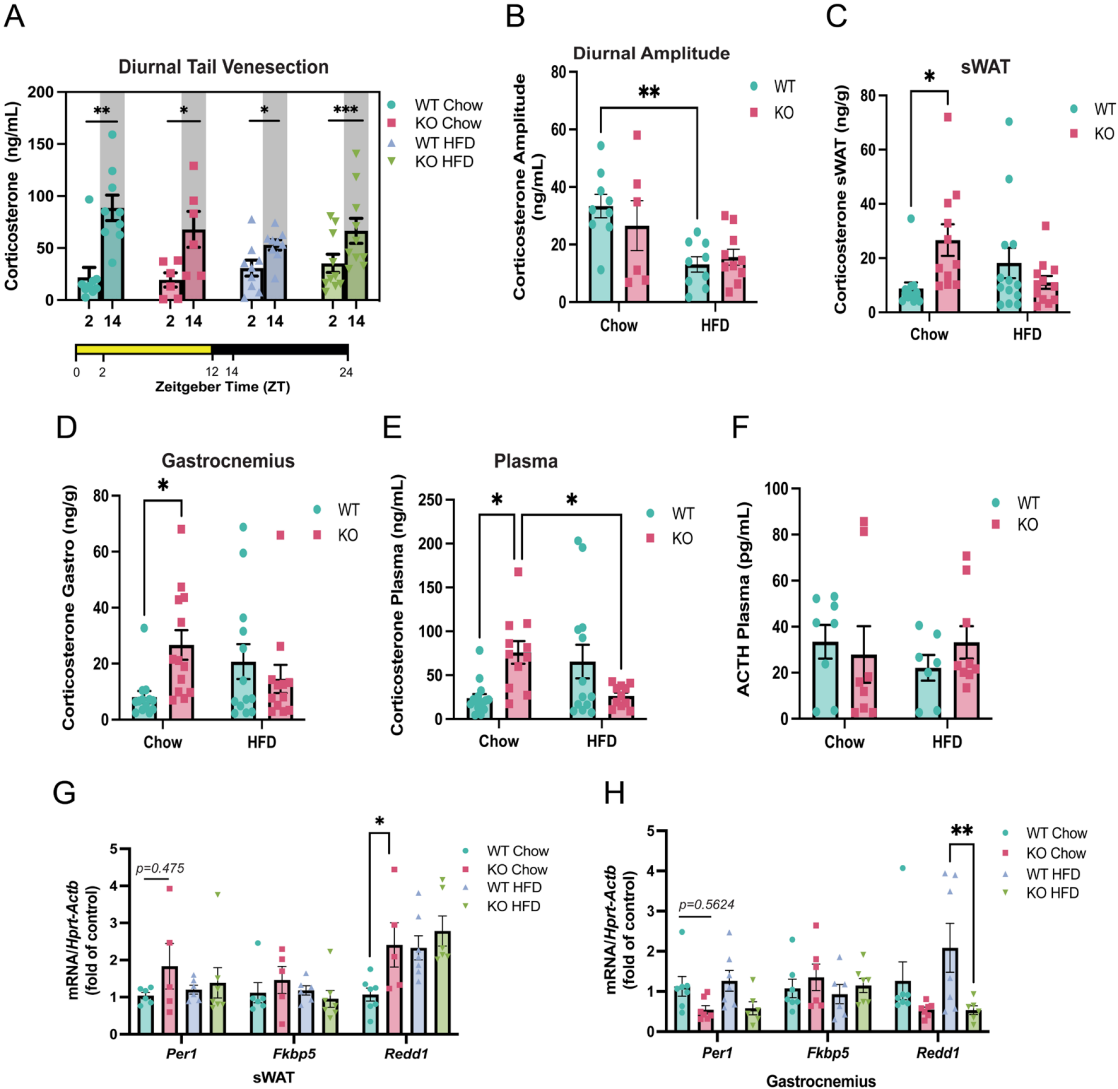
Deficiency of *Abcc1* induces accumulation of corticosterone in plasma, subcutaneous adipose tissue, and gastrocnemius muscle in lean but not obese mice. Steroid levels were evaluated by LC-MS/MS in plasma and tissue samples of wild-type (WT) and *Abcc1*-deficient (KO) male mice at week 7 and at the end of the study (week 9). (A) Corticosterone levels in plasma of mice obtained by tail venesection. Samples were collected at week 7 of the study, at 2 and 14 h after light onset (Zeitgeber time). (B) Quantification of corticosterone diurnal amplitude in (A) (diet: *P* < 0.01, genotype: *P* = 0.62, interaction: *P* = 0.29), *n* = 6–10, animals per group. Levels of corticosterone in terminal samples of (C) subcutaneous white adipose tissue (diet: *P*= 0.47, genotype: *P* = 0.22, interaction: *P* < 0.01), (D) gastrocnemius muscle (diet: *P* = 0.96, genotype: *P* = 0.21, interaction: *P* = 0.02) and (E) plasma (diet: *P* = 0.75, genotype: *P* = 0.59, interaction: *P* < 0.01), *n* = 8–13, animals per group. (F) Plasma levels of adrenocorticotropic hormone (ACTH) (diet: *P* = 0.73, genotype: *P* = 0.75, interaction: *P* = 0.34), *n* = 7–8, animals per group. (G) Evaluation of glucocorticoid-responsive genes (*Per1, Fkbp5* and *Redd1*) in subcutaneous white adipose tissue, and (H) gastrocnemius muscle by qRT-PCR (*n* = 6–7, animals per group). *P < 0.05, **P < 0.01. Data were analysed by two-way ANOVA with Tukey’s multiple comparisons test and expressed as mean ± s.e.m.

To determine if the metabolic phenotype in obese *Abcc1*-deficient mice was a result of increased tissue glucocorticoid action in key metabolic tissues, we assessed systemic and tissue glucocorticoid concentrations under both chow and HFD conditions. Assessment of diurnal corticosterone from tail-vein plasma showed no differences between genotypes (week 7, Fig. 2A and B). Dietary ‘flattening’ of the diurnal amplitude was observed in response to HFD, an effect that was more pronounced in WT (Fig. 2B). On chow diet, tissue corticosterone levels were increased in adipose tissue (Fig. 2C) and skeletal muscle (Fig. 2D) of KO mice compared to WT controls. Unexpectedly, quantification of plasma corticosterone in trunk blood collected at the time of tissue collection (week 9, Fig. 2E) revealed a striking similarity with tissue levels, with increased circulating corticosterone evident in KO mice compared to WT controls. These differences in tissue and plasma corticosterone between genotypes were abolished under HFD conditions. Interestingly, there were no differences in corticosterone levels in the liver, a tissue with negligible levels of Abcc1 (Supplementary Fig. 3B), supporting that the increments of corticosterone in adipose tissue and skeletal muscle could be indeed due to *Abcc1* actions. Further, to assess whether local corticosterone levels were influenced by the action of 11β-HDS1 we quantified tissue 11-dehydrocorticosterone but found similar patterns to corticosterone in both sWAT and gastrocnemius (data not shown), indicating this enzyme is unlikely to play a role here.

To explore the basis for altered corticosterone levels, we assessed HPA axis activation by measuring plasma ACTH but found no significant difference between genotypes under either dietary condition (Fig. 2F). To assess clearance of corticosterone, in a separate experiment, we infused adrenalectomised male WT and *Abcc1*-deficient mice with corticosterone under chow diet conditions to achieve steady-state plasma concentrations; however, there were no differences in steady-state exogenous corticosterone levels between genotypes (Supplementary Fig. 2A, B and C). To determine if KO mice exhibited a heightened stress response, we performed an acute restraint stress test in male WT and *Abcc1-*deficient mice, again showing no significant differences in corticosterone response between genotypes (Supplementary Fig. 3A).

To determine if tissue glucocorticoid action mirrored the changes in tissue corticosterone levels, we assessed several known glucocorticoid-responsive transcripts in adipose tissue (Fig. 2G) and skeletal muscle (Fig. 2H). In sWAT, the glucocorticoid-responsive gene *Redd1* (Kim *et al.* 2023) was increased in KO animals under chow conditions, but no differences were observed under HFD conditions (Fig. 2G). In skeletal muscle, neither *Per1, Fkbp5* nor *Redd1* were elevated in KO mice, and indeed *Redd1* was paradoxically lower in KO than WT mice on HFD (Fig. 2H).

In the absence of elevated tissue glucocorticoid levels and glucocorticoid-regulated transcripts in adipose or skeletal muscle in *Abcc1*-deficient mice on HFD, we explored other mechanisms that might explain their adverse glucose metabolism phenotype.

### Transcriptomic and proteomic analyses reveal differential responses to high-fat diet in adipose tissue from *Abcc1*-deficient mice

**Figure 3:**
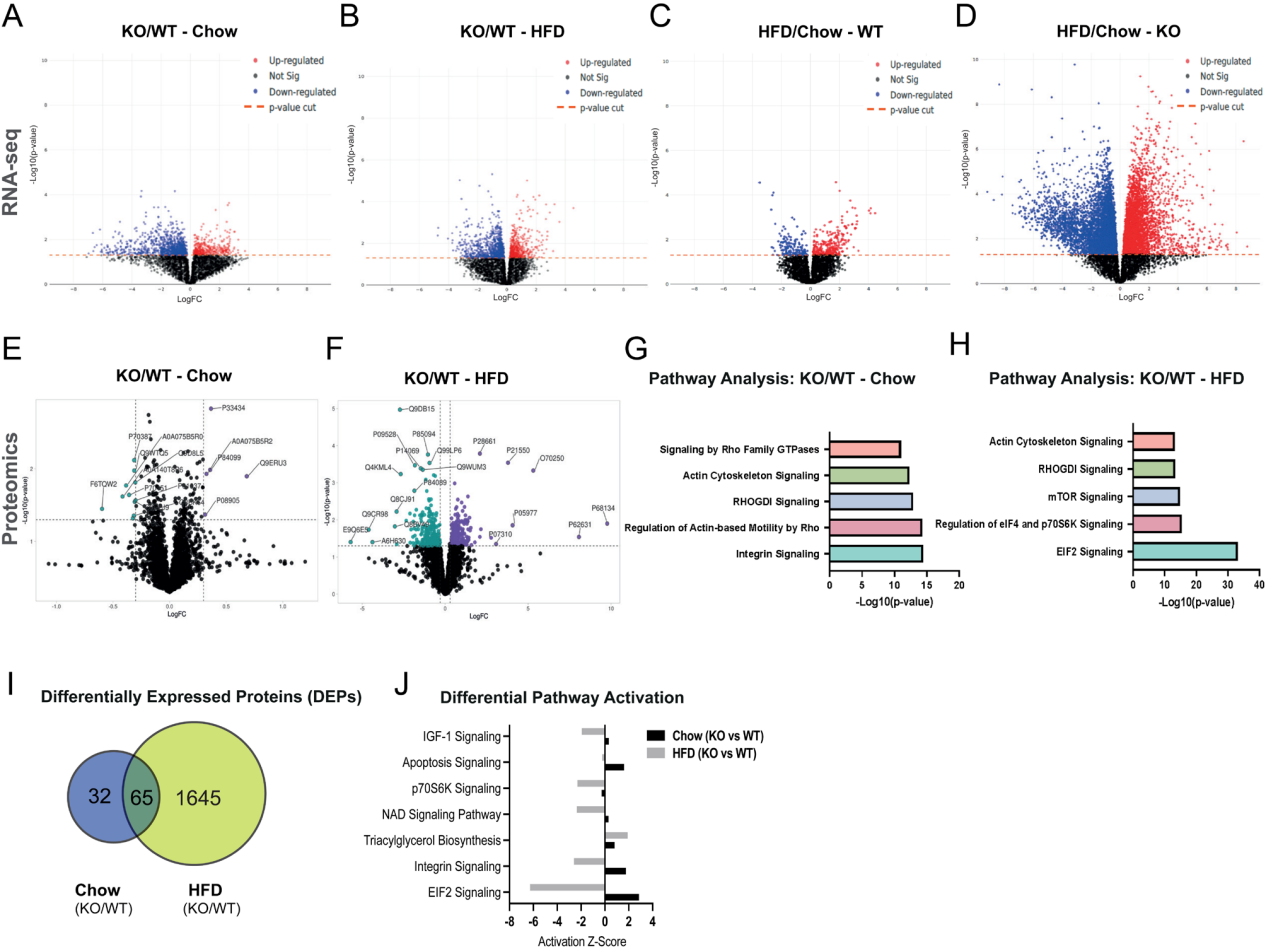
Transcriptomic and proteomic analyses of subcutaneous adipose tissue reveal an amplified impact of HFD in *Abcc1*-deficient mice. (A) Volcano plot showing the log10 transformed unadjusted *P*-values against log2 fold change of all the genes identified in sWAT between wild-type (WT) and *Abcc1*-deficient (KO) mice fed with chow diet or (B) HFD during 9 weeks, and between HFD and chow diet in (C) WT and (D) KO mice. (E) Volcano plot showing the differential expression analysis of proteins in sWAT between WT and KO mice fed with control diet (chow) or (F) HFD for 9 weeks. Proteins differentially expressed are shown in purple (upregulated) and green (downregulated). (G) Ingenuity pathway analysis (IPA) of differentially expressed proteins (DEPs) in sWAT of WT and KO mice fed chow diet and (H) HFD. The *x*-axis indicates −log10 of the *P-*value, and *y*-axis indicates the corresponding canonical pathways. (I) Venn diagram showing proteins included in the IPA analysis in J, with differential expression (FC + 2) in KO compared with WT in response to the diets. (J) Comparative analysis of differential activation of pathways identified in KO vs WT mice under chow and HFD conditions. The *x*-axis indicates *Z*-score explaining activation of the pathways on the *y*-axis. Both omics analyses were performed in four animals per experimental group.

We performed bulk RNA sequencing (RNA-Seq) analysis in sWAT from WT and *Abcc1*-deficient mice. Differential expression analysis comparing genotype effects revealed a number of differentially expressed genes (DEGs) under both chow (Fig. 3A) and HFD (Fig. 3B) conditions. However, following adjustment for multiple comparison testing, significant DEGs (adjusted *P*-value < 0.05) between genotypes were no longer observed in either chow or HFD mice (Supplementary Fig. 4A and B). A comparison of dietary effects also revealed a number of DEGs in both genotypes, with the effect exacerbated in KO mice (Fig. 3C and D). Following adjustment for multiple comparison testing, 6517 DEGs were identified in *Abcc1*-deficient mice between HFD and chow diet versus 16 in WT mice.

We evaluated whether genes that were differentially expressed in *Abcc1*-deficient mice were likely to reflect altered glucocorticoid signalling. We identified genes in sWAT that have previously been shown to be regulated by glucocorticoids both in mice (following dexamethasone treatment) and in humans (inferred from trans-QTL analyses for genes associated with variation in plasma cortisol) (Bankier *et al.* 2023). These included *Pkp2, Osmr, Phyh, Zc3h7b* and* Me2*; none of these genes were differentially expressed when comparing sWAT gene expression in KO vs WT mice.

We also undertook a complementary proteomic approach. Comparison of genotype effect under individual dietary conditions identified a number of differentially expressed proteins (DEPs) (Fig. 3E and F). Ingenuity pathway analysis (IPA) was used to identify enriched biological pathways amongst the DEPs and infer potential regulatory mechanisms under each dietary condition. The results show that in lean, chow-fed animals, the top dysregulated pathways in KO mice were related to tissue remodelling and trafficking of vesicles (Fig. 3G). In obese, HFD-fed mice, the most dysregulated pathways in *Abcc1-*deficient mice were associated with reticular stress, mTOR signalling and tissue remodelling (Fig. 3H). Comparative analysis across both genotype and diet identified DEPs between *Abcc1-*deficient and WT mice on HFD as a change from the chow diet (Fig. 3I), and subsequent pathway activation *Z-*scores identified differential activation of signalling pathways related to EIF2, integrin, IGF-1, S6K, apoptosis and NAD in KO mice under chow and HFD conditions (Fig. 3J).

### Transcriptomic and proteomic analyses identify impaired oxidative phosphorylation in skeletal muscle of *Abcc1*-deficient mice

**Figure 4:**
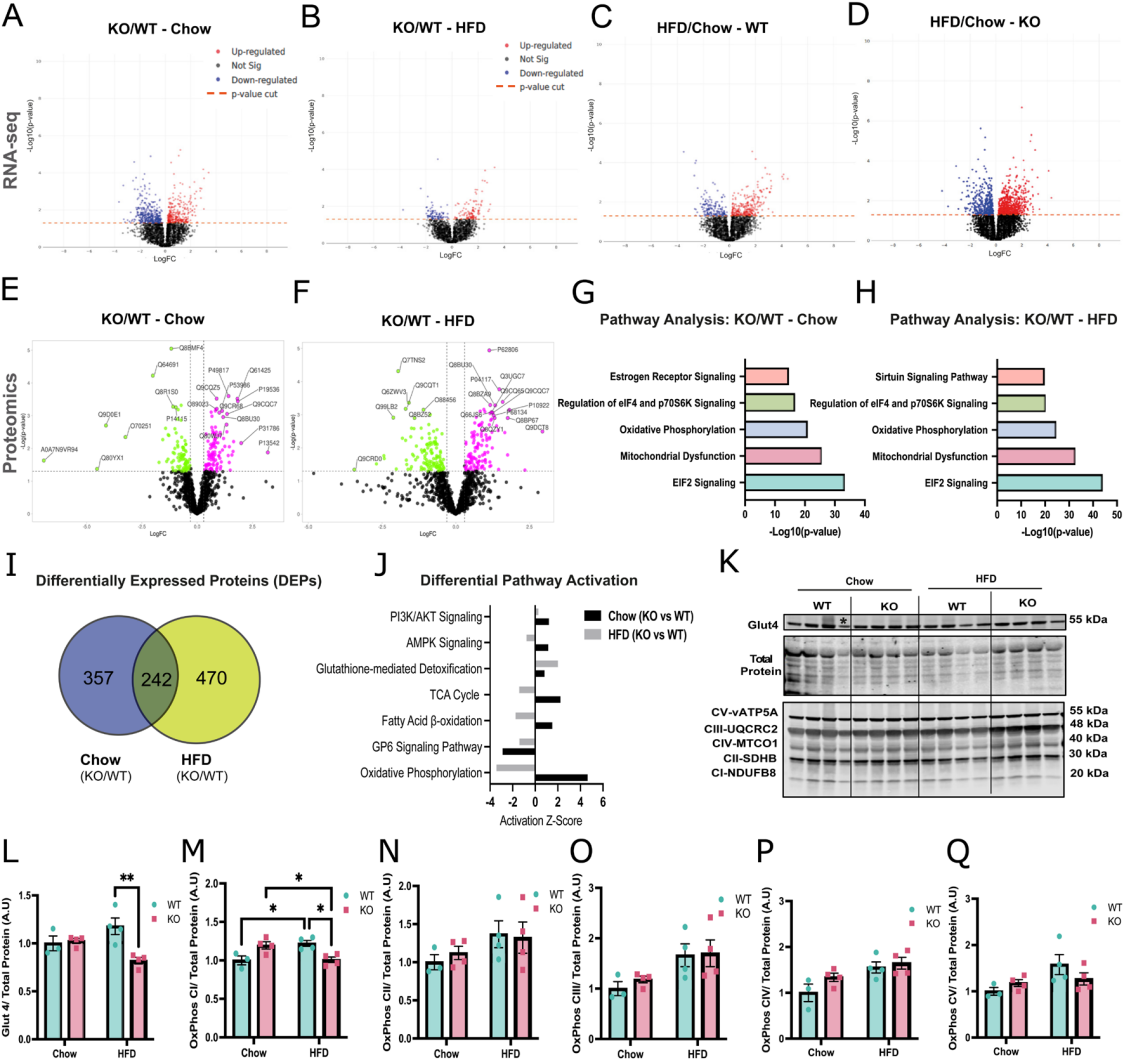
Proteomic and pathway analyses of *Abcc1*-deficient mice in gastrocnemius muscle reveal impairment in oxidative phosphorylation in mice exposed to HFD. (A) Volcano plot showing log10 transformed unadjusted *P*-values against log2 fold change of all the genes identified in gastrocnemius muscle between WT and *Abcc1* KO mice fed with chow diet or (B) HFD for 9 weeks, and between HFD and chow diet in (C) WT and (D) KO mice. (E) Volcano plot showing the differential expression analysis in gastrocnemius between WT and *Abcc1* KO mice fed chow and (F) HFD during 9 weeks. Proteins differentially expressed are shown in fuchsia (upregulated) and green (downregulated). (G) Ingenuity pathway analysis (IPA) of differentially expressed proteins in gastrocnemius muscle of WT and *Abcc1* KO mice fed with control (chow) diet or (H) HFD. The *x*-axis indicates −log10 of the *P*-value, and the *y*-axis indicates the corresponding canonical pathways. (I) Venn diagram showing proteins included in the IPA analysis in J, with differential expression (FC + 2) in KO compared with WT in response to the diets. (J) Comparative analysis of differential activation on pathways identified in KO versus WT mice under chow and HFD conditions. The *x*-axis indicates *Z*-score explaining the activation of the pathways in the *y*-axis. The omics analyses were performed in four animals per experimental group. (K) Western blot analysis (gastrocnemius) and (M–Q) densitometric quantification of (L) Glut4 and OXPHOS: complex I (M), complex II (N), complex III (O), complex IV (P), and complex V (Q). Data were normalised by the staining of total proteins (*n* = 3–4, animals per group). (*) Rout method (*Q* = 1%) was used to identify outliers. *P < 0.05, **P < 0.01. Data were evaluated by two-way ANOVA with Tukey’s multiple comparisons test and are expressed as mean ± s.e.m.

Similar to sWAT, transcriptomic analysis of skeletal muscle revealed DEGs between *Abcc1*-deficient and WT mice on both chow (Fig. 4A) or HFD (Fig. 4B). However, after adjusting for multiple comparison testing, no significant (adjusted *P*-value < 0.05) DEGs were observed between genotypes on either chow or HFD (Supplementary Fig. 4C and D). In contrast to the more striking effect of HFD observed in the adipose tissue of KO vs WT mice, skeletal muscle showed a more modest response to HFD (Fig. 4C and D). However, the transcriptional response to HFD was still exaggerated in *Abcc1-*deficient mice compared with WT mice in skeletal muscle (five DEGs in KO mice vs 0 in WT mice).

As was observed in adipose tissue, proteomic analyses comparing genotypes under each dietary condition identified DEPs between KO and WT mice under both chow (Fig. 4E) and HFD (Fig. 4F). IPA analysis revealed increased enrichment amongst the DEPs of proteins in pathways associated with reticular stress, mitochondrial dysfunction, and oxidative phosphorylation under chow and HFD conditions (Fig. 4G and H). Comparative analysis across both genotype and diet identified DEPs between *Abcc1-*deficient and WT mice on HFD as a change from the chow diet (Fig. 4I), and differential activation of pathways in KO mice compared to WT mice under dietary conditions, with pathways related to the TCA cycle, β-oxidation, and oxidative phosphorylation activated under chow diet, but inactivated under HFD conditions (Fig. 4J).

To validate the inferred changes in glycolysis and oxidative phosphorylation (Fig. 4K), we used Western blots to assess the protein levels of components of oxidative phosphorylation complex machinery (OXPHOS), and Glut4, a key glucose transporter in skeletal muscle (Fig. 4L, M, N, O, P and Q). Western blots revealed decreased levels of Glut4 and complex I in KO mice under HFD.

## Discussion

Our prior work shows that *Abcc1* acts as a transporter of exogenously administered glucocorticoid in adipose tissue (Nixon *et al.* 2016). Here, we tested whether *Abcc1* has a role as a transporter of endogenous glucocorticoid in adipose tissue and skeletal muscle and whether it influences adiposity and glucose metabolism. Although tissue glucocorticoid levels were elevated with *Abcc1* deficiency in adipose tissue and skeletal muscle, these differences could not be dissociated from simultaneous differences in plasma glucocorticoid levels and were not sustained when mice were given a HFD. Moreover, analysis of transcriptomic and proteomic data in adipose tissue and skeletal muscle did not provide evidence for the enrichment of glucocorticoid-responsive genes amongst those that differed with *Abcc1* deficiency. Indeed, despite increased tissue corticosterone levels in lean *Abcc1-*deficient mice, *Redd1*, a glucocorticoid-responsive gene (Kim *et al.* 2023), was simultaneously increased in sWAT but not trending towards being decreased in gastrocnemius muscle, suggesting a transcriptional regulation in muscle over and above that by glucocorticoids. However, despite the lack of evidence for enhanced glucocorticoid action, *Abcc1* deficiency caused metabolic dysfunction and a greater transcriptional and proteomic response to HFD. This suggests that, in contrast with our hypothesis, there is a protective metabolic effect of *Abcc1* which is glucocorticoid-independent.

We observed changes in plasma corticosterone with *Abcc1* deficiency in terminal trunk blood samples in lean mice but not in diurnal tail-nick samples. This discrepancy alluded to a greater ‘stress response’ during sacrifice in *Abcc1*-deficient mice. However, when we tested this formally using an acute restraint test, we were unable to demonstrate a difference. Previous studies suggested that ABCC1 influences glucocorticoid clearance (Livingstone *et al.* 2014, 2017), but we did not find evidence for this when we infused corticosterone in adrenalectomised mice. There is also evidence that ABCC1 influences HPA axis negative feedback in humans (Kyle *et al.* 2022) but we did not find differences in plasma ACTH to substantiate a central activation of the HPA axis, albeit that measurements of ACTH are labile and hence insensitive to assess changes in the HPA axis (Toprak *et al.* 2016, Wu & Xu 2017, Donegan *et al.* 2019). Together, this suggests that differences in stress-induced HPA activation or glucocorticoid clearance are not responsible for the corticosterone phenotype observed in lean *Abcc1*-deficient mice. This unexpected phenomenon of higher plasma corticosterone during terminal sampling represents a confounder in the interpretation of endogenous tissue corticosterone levels in lean *Abcc1*-deficient mice that we have been unable to overcome. However, this effect appears to be over-ridden by the well-documented effect of HFD to alter HPA axis function (Morton *et al.* 2005). The absence of elevated tissue glucocorticoid levels in *Abcc1*-deficient mice on HFD, along with the evidence that HFD did not alter *Abcc1* expression in our experiments, suggests that ABCC1 does not have a potent effect on tissue levels of endogenous corticosterone.

Even in the absence of altered tissue glucocorticoid levels, or changes in adiposity, metabolic assessments revealed impaired glucose tolerance and hyperinsulinaemia in *Abcc1-*deficient mice under HFD. In light of this paradox, we sought to study key differences in the transcriptome and proteome in the adipose tissue and skeletal muscle of *Abcc1-*deficient mice, which may be independent of glucocorticoid action and might mediate the adverse metabolic response (Calejman *et al.* 2022, Cottam *et al.* 2022). It is worthy of note that bulk RNA-seq can have some pitfalls when the heterogeneity of the tissues is high (Li & Wang 2021, Denninger *et al.* 2022, Noureen *et al.* 2022). An expansion of adipose tissue due to HFD exposure has been shown not only to change the cell profile in adipose tissue by increasing proinflammatory cells but also in skeletal muscle (Fuster *et al.* 2016, Chait & den Hartigh 2020, Morgan *et al.* 2020, Miranda *et al.* 2023). Despite these limitations, in white adipose tissue, both mRNA and protein profiling revealed that *Abcc1* deficiency amplifies the response to HFD. Key signalling pathways that were differentially regulated include extracellular matrix (ECM) and reticular stress. A link between ECM and ABCC1 was described in HT-29 cells, where the ECM from tumour cells was able to upregulate *ABCC1*, increasing their chemoresistance capacity (Hoshiba & Tanaka 2016). We also observed the Eukaryotic Initiation Factor 2 (eIF2) signalling pathway to be less active in *Abcc1*-deficient mice under HFD. eIF2 is a key protein regulating reticular stress, a process that has been demonstrated to be a major contributor to the metabolic dysfunction induced by obesity (Han & Kaufman 2016, Li *et al.* 2020).

In skeletal muscle, the proteomic analysis shed light on pathways related to glucose homeostasis that could explain the impairment of glucose tolerance and hyperinsulinaemia in the *Abcc1*-deficient mice receiving HFD. These included mitochondrial dysfunction, oxidative phosphorylation, and reticular stress, all fundamental to metabolism (Rani *et al.* 2016, Antonopoulos & Tousoulis 2017, Zhu *et al.* 2022). Supporting the inference of impaired oxidative phosphorylation in skeletal muscle with *Abcc1* deficiency, our assessment of the OXPHOS complex identified decreased protein levels in complex I, the initiator of the respiratory chain (Sharma *et al.* 2009). Further, diminished levels of Glut4 in skeletal muscle. Overall, these changes could plausibly mediate the observed metabolic effects of *Abcc1* deficiency.

A limitation of our studies is that we focused on subcutaneous WAT, in line with previous work demonstrating a role for ABCC1 in modulating exogenous glucocorticoid levels (Nixon *et al.* 2016). We did observe a reduction in (BAT weight in lean *Abcc1*-deficient mice but not in mice fed an HFD. We have not pursued this observation further here, although it is notable that a recent study showed BAT as the most sensitive adipose tissue depot during acute (1 week) corticosterone administration (Harvey *et al.* 2023).

Although we have not determined the mechanism by which ABCC1 would influence the metabolic pathways that are implicated in white adipose tissue and skeletal muscle, our work has identified tissue-specific pathways that provide a stronger platform for establishing the mechanism. ABCC1 is a multidrug resistance protein that acts on the efflux not only of glucocorticoids but also a variety of xenobiotics (Cole 2014, Devine *et al.* 2023). Endogenous substrates of ABCC1 include estradiol-17β-glucuronate (Slot *et al.* 2008, Cole 2014, Devine *et al.* 2023), proinflammatory molecules (LTC4), antioxidants (GSH), and signalling lipids (SP1) (Nieuwenhuis *et al.* 2009, Cole 2014, Devine *et al.* 2023). Many of these substrates are altered in obesity (Fuster *et al.* 2016, Antonopoulos & Tousoulis 2017, Cottam *et al.* 2022) and could mediate the worsened metabolic profile in *Abcc1-*deficient mice under HFD. Our findings of a novel role for ABCC1 in limiting the adverse metabolic effects of obesity should stimulate further investigation of the various substrates of ABCC1 and their transmembrane transport in obesity.

## Supplementary Materials

Supplementary Material

Figure S1. Glucose, insulin tolerance test and Abcc1 expression in tissues

Figure S2. Glucocorticoid Clearance

Figure S3. Acute restraint test, corticosterone levels and glucocorticoid responsive genes in liver.

Figure S4. Transcriptomics and proteomics adjusted p-value in sWAT and skeletal muscle

## Declaration of interest

All the authors declare no conflict of interest, financial or otherwise. Ruth Andrew is the Co-Editor-in-Chief of the *Journal of Endocrinology*. Ruth Andrew was not involved in the review or editorial process for this paper, on which she is listed as an author.

## Funding

Supported by a Wellcome Senior Investigator Award (to BRW), an Early Career Grant from the Society for Endocrinologyhttp://dx.doi.org/10.13039/501100000382 (to EV), and a BHF Intermediate Fellowship Award (to MN). TMW would like to acknowledge support from the BBSRC ISP1 BBS/E/RL/230001C and core capability funding. For the purpose of open access, the author has applied a Creative Commons Attribution (CC BY) licence to any Author Accepted Manuscript version arising from this submission.

## Data availability

Data are available upon request or from the University of Edinburgh Data Store https://datashare.ed.ac.uk.

## Author contribution statement

EV, BRW and MN conceived and designed the studies; EV, AM, RAM, LI and JA performed experiments; EV, RAK, MP, JPS and DK analysed data; EV, NZH, BRW and MN interpreted results; EV prepared figures and drafted the manuscript; EV, BRW, RAM, JPS, NM, RA, RS, TMW and MN edited and revised the manuscript; all authors approved the final version of the manuscript.

## Acknowledgements

The authors thank the Mass Spectrometry Core at the Queen’s Medical Research Institute (QMRI), University of Edinburgh, and the Edinburgh Metabolic Phenotyping Facility, University of Edinburgh, for the skilled help and support. We thank Dr Sean Bankier for his guidance on the glucocorticoid-regulated gene network.
