# Supplementary material for: ATP-binding cassette family C member 1 constrains metabolic responses to high-fat diet in male mice
Source: J Endocrinol. 2024 Jul 3;262(2):e240024. doi: 10.1530/JOE-24-0024 (PMC11301423; doi:10.1530/JOE-24-0024)
Supplement: Supplementary Material [file supplementary_material.pdf]

JOURNAL OF ENDOCRINOLOGY

SUPPLEMENTARY MATERIALS

ATP-Binding Cassette Family C member 1 constrains metabolic responses to high-fat diet in male mice

Elisa Villalobos[1,4], Allende Miguelez-Crespo[1], Ruth A. Morgan[1,2], Lisa Ivatt[1], Mhairi Paul[1], Joanna P. Simpson[1], Natalie Z. M. Homer[1], Dominic Kurian[3], Judit Aguilar[3], Rachel A. Kline[3], Thomas M. Wishart[3], Nicholas Morton[1,5], Roland H. Stimson[1], Ruth Andrew[1], Brian R. Walker[1,4] & Mark Nixon[1]*

[1] British Heart Foundation Centre for Cardiovascular Science, The Queen's Medical Research Institute, University of Edinburgh, Edinburgh, United Kingdom.

[2] Scotland's Rural College, The Roslin Institute, Easter Bush Campus, United Kingdom.

[3] The Roslin Institute, Royal (Dick) School of Veterinary Studies, College of Medicine and Veterinary Medicine, University of Edinburgh, Easter Bush, United Kingdom.

[4] Translational and Clinical Research Institute, Newcastle University, Newcastle upon Tyne, United Kingdom.

5Centre for Systems Health and Integrated Metabolic Research, Nottingham Trent University, Nottingham, United Kingdom

Correspondence: Mark Nixon (M.Nixon@ed.ac.uk)

MATERIALS AND METHODS

Adrenalectomy and Mini-pump Insertion

Adult male mice (8-10- weeks old, n=7-8 per group), were bilaterally adrenalectomised under anaesthesia and the subcutaneous mini-pump inserted dorsally at the same procedure using the same opening. Muscle wall was closed with absorbable sutures and skin incisions closed with stainless steel clips. Mice were daily monitored and weight after surgery, to ensure recovery. Mice were single housed animals after surgery and 0.9% saline drinking water was provided.

Pump details: osmotic mini-pumps (Alzet #2001) containing steroid (corticosterone and cortisol; 10.4mg/ml each, in propylene glycol) was prepared the day before the study and primed in saline solution overnight at 37°C. The saline priming solution was changed an hour before pump insertion. The pump was in situ for 7 days. Mice were culled between 9:00 to 11:30AM by decapitation, and the trunk blood and tissues were collected to perform steroid measurements. All the procedures were performed under UK Home Office license and approved by the University of Edinburgh, Bioresearch & Veterinary Services (BVS).

Acute Restraint Test

Adult male mice (8-10- weeks old, n=8 per group), underwent acute restraint stress, by keeping the mice in a tube rodent holder for 10 min. Blood was taken by tail venesection at basal conditions (immediately prior to stressor), 10 minutes (immediately post-stressor), and 90 minutes after stressor to assess return to baseline. Corticosterone was measured by LC-MS/MS. All the procedures were performed under UK Home Office license and approved by the University of Edinburgh, Bioresearch & Veterinary Services (BVS).

FIGURE LEGENDS

Supplementary Figure 1. Glucose and insulin tolerance test raw glucose values, and *Abcc1* expression in tissues.

Animals were weaned onto chow diet from 4-weeks of age and either chow diet or HFD feeding started at 8- to 10-weeks of age and continued for 9 weeks. **(A)** Basal glucose levels after 5-6 hours of fast **(B)** Intraperitoneal insulin tolerance test (IP-ITT) performed at week 7 of the study in WT and KO mice fed with chow diet and HFD (Time: $p < 0.01$, Experimental group: $p = 0.19$, Interaction: $p = 0.09$). **(C)** quantification of area over the curve (AOC) in (A) (Diet: $p = 0.52$, Genotype: $p = 0.09$, Interaction: $p = 0.48$), n=4-7 animals per group. **(D)** Evaluation of *Abcc1* gene expression in subcutaneous white adipose tissue, and **(E)** gastrocnemius muscle by qRT-PCR (n=5-6, animals per group). Statistical analysis was done by Two-Way ANOVA with repeated measures (B), followed by a *post-hoc* test (*Tukey*). A *p-value* < 0.05 was considered significant. Data are expressed as mean ± SEM.

Supplementary Figure 2. Glucocorticoid Clearance

Adult male *Abcc1*-deficient (KO) mice and WT littermates (7-8 per group) were bilaterally adrenalectomized and subcutaneous mini-pumps inserted to deliver corticosterone and cortisol (10.4 mg/mL each) for 7 days. Steroid levels were evaluated in terminal samples by LC-MS/MS in plasma **(A)**, subcutaneous white adipose tissue (sWAT) **(B)**, and gastrocnemius muscle (gastroc) **(C)**. **p-value* < 0.05, by Student's t-test between WT vs. KO mice. Data are expressed as mean ± SEM.

Supplementary Figure 3. Acute restraint test and analysis of hepatic corticosterone levels and glucocorticoid responsive transcripts after diet.

(A) Male, mice of 8- to 10-weeks of age, underwent acute restraint stress (in a tube for 10min), blood taken by tail venesection at Time=0 (immediately prior to stressor), 10 minutes (immediately post-stressor), and 90 minutes after stressor (n=8, animals per group). Animals

were fed either chow diet or HFD feeding started at 8- to 10-weeks of age and continued for 9 weeks. **(B)** Steroids levels were evaluated by LC-MS/MS in liver of WT and KO male mice at the end of the main study (9 weeks chow or HFD) (n=5-8, animals per group). **(C)** Evaluation of glucocorticoid-responsive genes *(Pepck1, Fkbp5, Per1 and Lpl)* in liver by qRT-PCR (n=5-6, animals per group). $p < 0.05$ was considered significant. Repeated measures ANOVA (A) and two-way ANOVA with Tukey's multiple comparisons test (B-C). Data are expressed as mean + SEM.

Supplementary Figure 4. Transcriptomic and Proteomic adjusted p-value in sWAT and skeletal muscle.

(A-D) Volcano plots showing the log10 transformed adjusted p-values against log2 fold change of all the genes identified in sWAT **(A-B)** and skeletal muscle **(C-D)** between wild-type (WT) and *Abcc1*-deficient (KO) mice fed with chow diet or HFD during 9 weeks, respectively. **(E-H)** Volcano plots showing the log10 transformed adjusted p-values against log2 fold change of the differential expression analysis of proteins in sWAT **(E-F)** and skeletal muscle **(G-H)** between wild-type and *Abcc1* knockout mice fed with control diet (chow) or HFD for 9 weeks, respectively.
