# Supplementary figures and images for: ATP-binding cassette family C member 1 constrains metabolic responses to high-fat diet in male mice
Source: J Endocrinol. 2024 Jul 3;262(2):e240024. doi: 10.1530/JOE-24-0024 (PMC11301423; doi:10.1530/JOE-24-0024)

**Figure S1. Glucose, insulin tolerance test and Abcc1 expression in tissues**

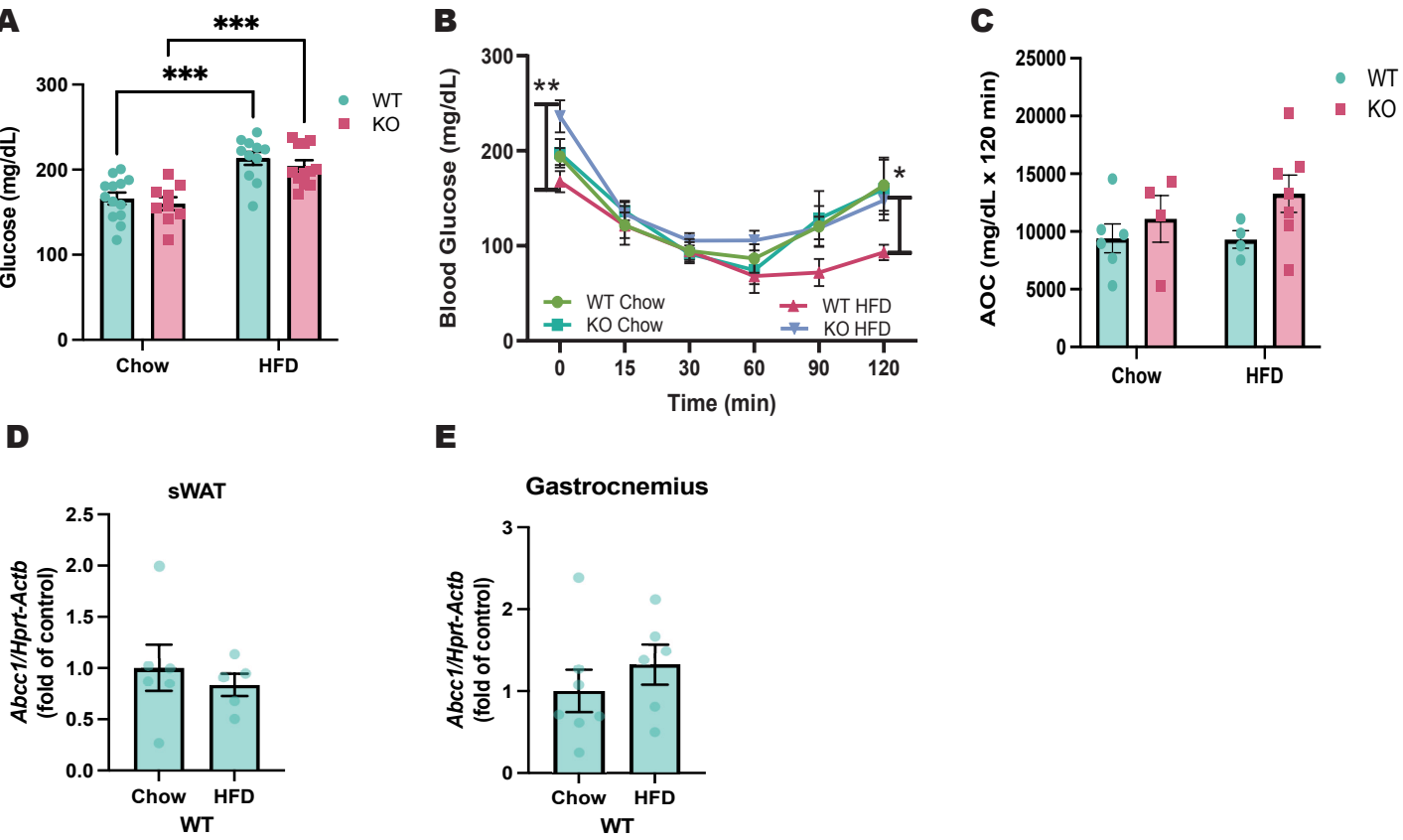

Supplement: Figure S1. Glucose, insulin tolerance test and Abcc1 expression in tissues [file supplementary_figure_1.pdf]

Figure S2. Glucocorticoid Clearance

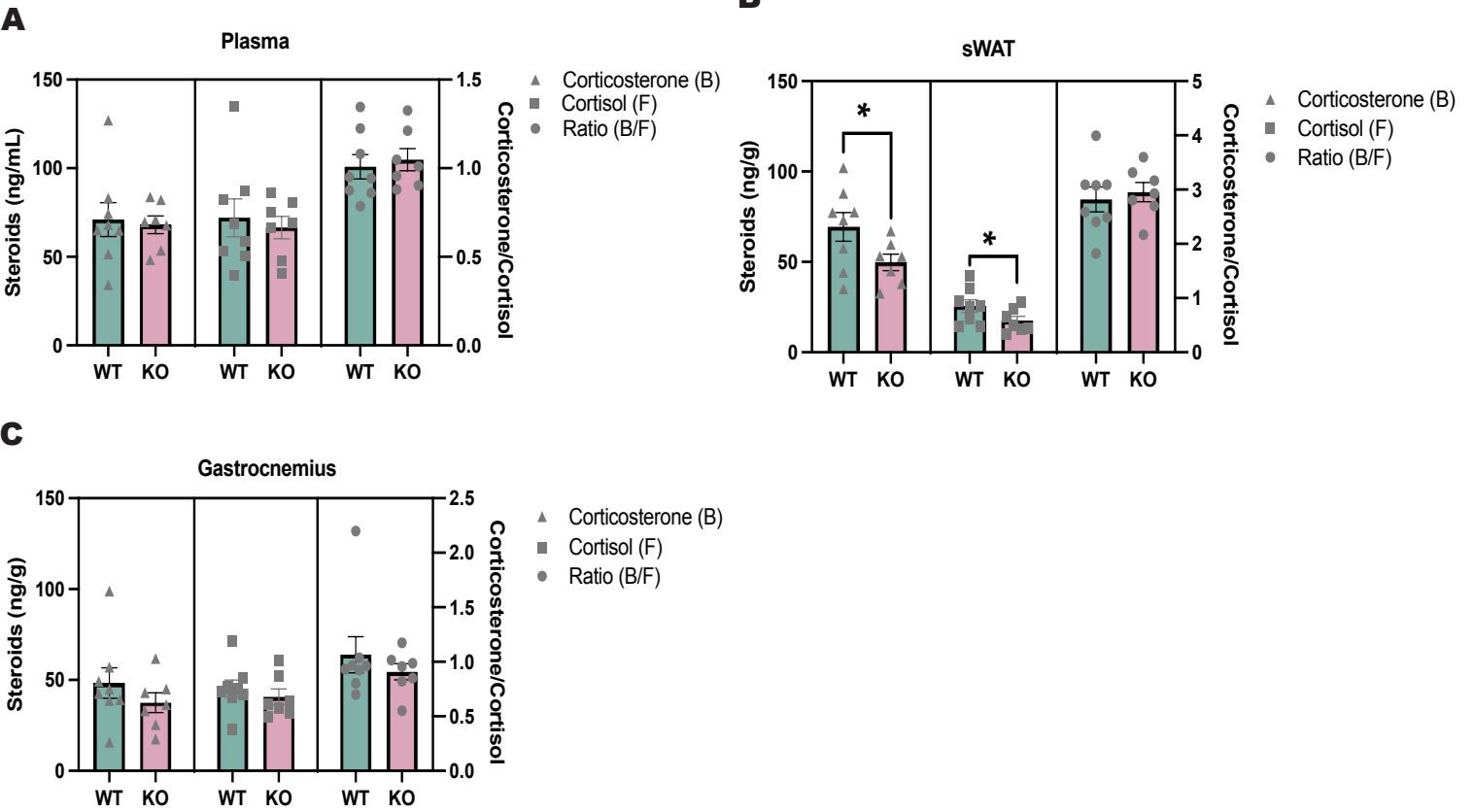

Supplement: Figure S2. Glucocorticoid Clearance [file supplementary_figure_2.pdf]

**Figure S4. Transcriptomics and proteomics adjusted p-value in sWAT and skeletal muscle**

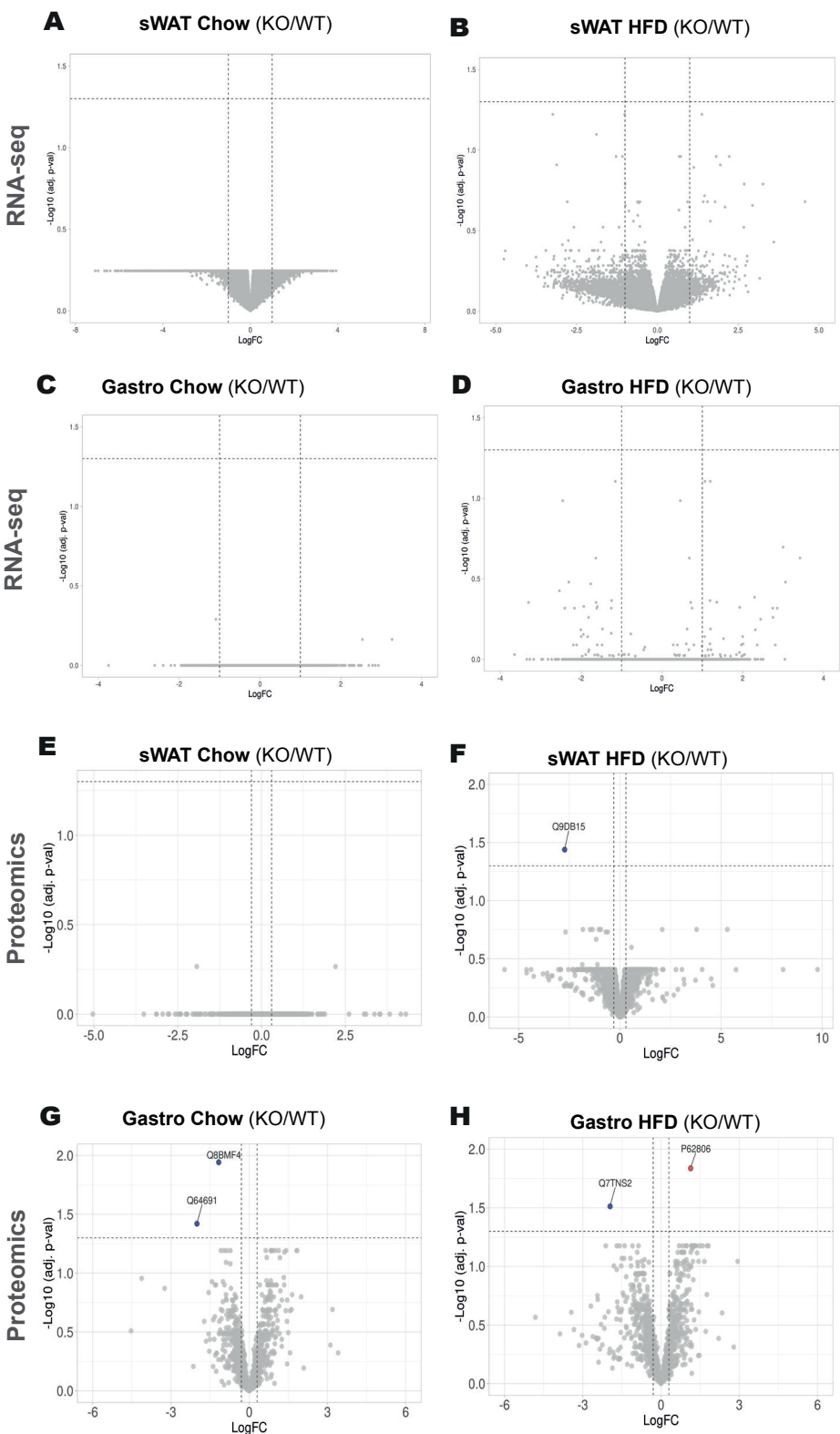

Supplement: Figure S4. Transcriptomics and proteomics adjusted p-value in sWAT and skeletal muscle [file supplementary_figure_4.pdf]
