# Supplementary material for: ATP-binding cassette family C member 1 constrains metabolic responses to high-fat diet in male mice
Source: J Endocrinol. 2024 Jul 3;262(2):e240024. doi: 10.1530/JOE-24-0024 (PMC11301423; doi:10.1530/JOE-24-0024)

Figure S3. Acute restraint test, corticosterone levels and glucocorticoid responsive genes in liver.

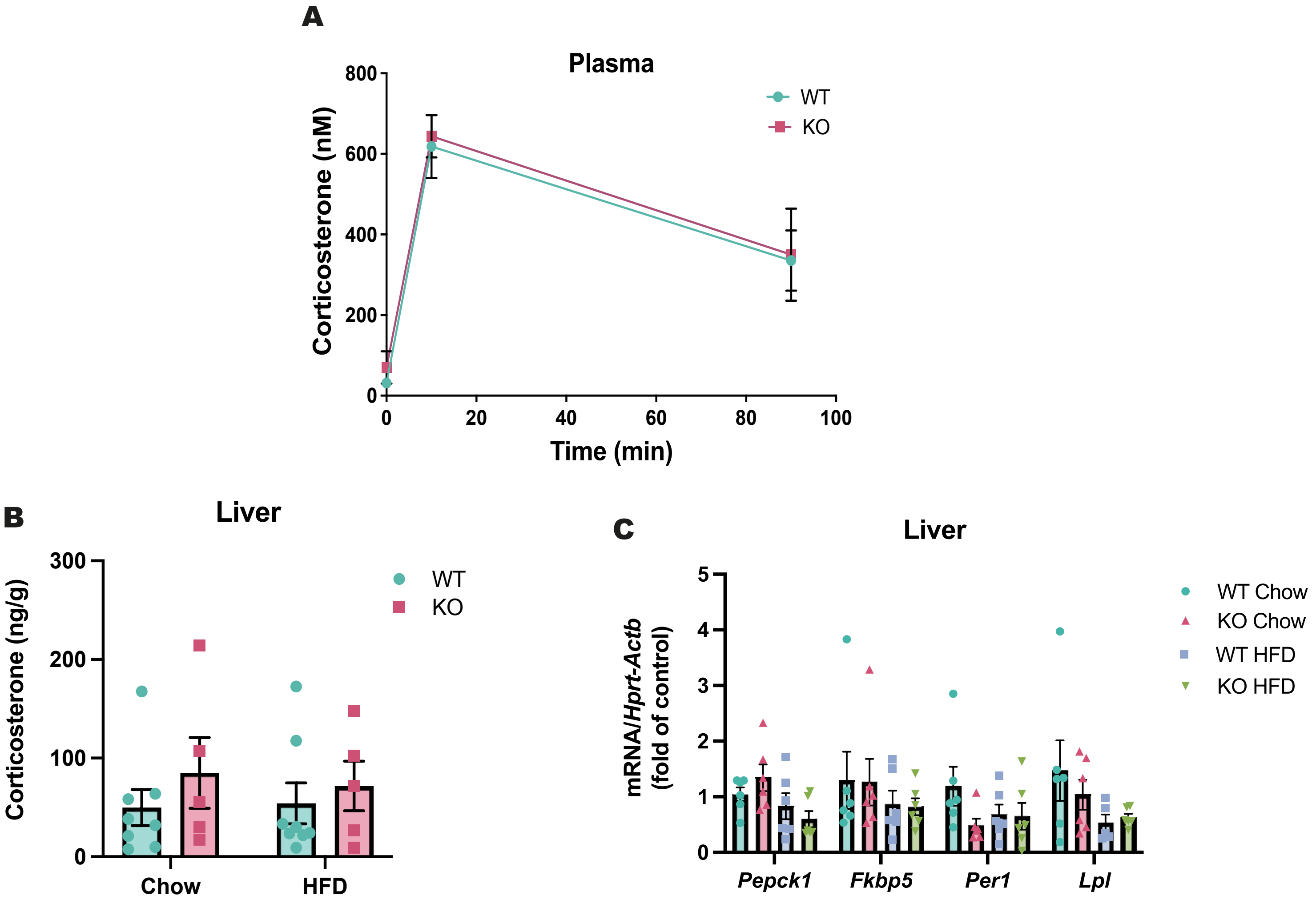

Supplement: Figure S3. Acute restraint test, corticosterone levels and glucocorticoid responsive genes in liver. [file supplementary_figure_3.pdf]
